# Evolution and molecular bases of reproductive isolation

**DOI:** 10.1016/j.gde.2022.101952

**Published:** 2022-07-16

**Authors:** G Ozan Bozdag, Jasmine Ono

**Affiliations:** 1 School of Biological Sciences, Georgia Institute of Technology, Atlanta, GA 30332, USA; 2 Centre for Life’s Origins and Evolution, Department of Genetics, Evolution and Environment, University College London, London WC1E 6BT, UK

## Abstract

The most challenging problem in speciation research is disentangling the relative strength and order in which different reproductive barriers evolve. Here, we review recent developments in the study of reproductive isolation in yeasts. With over a thousand genome-sequenced isolates readily available for testing the viability, sterility, and fitness of both intraspecies and interspecies hybrid crosses, *Saccharomyces* yeasts are an ideal model to study such fundamental questions. Our survey demonstrates that, while chromosomal-level mutations are widespread at the intraspecific level, anti-recombination-driven chromosome missegregation is the primary reproductive barrier between species. Finally, despite their strength, all of these postzygotic barriers can be resolved through the asexual life history of hybrids.

## Introduction

Reproductive isolation is interesting to biologists because it gets at the fundamental question: what is a species and how is it formed? Reproductive isolation (RI) allows for the maintenance of diversity and forms the basis of the Biological Species Concept. While it is not the only thing that defines species, we find it useful because it generally correlates with sequence divergence at both the individual–gene and at the whole-genome level, at least in yeasts [1].

RI in yeasts comes from many sources and, interestingly, often differs by whether we are investigating species or populations, and wild or domesticated strains. Here, we describe recent findings in the study of RI in yeasts (for more comprehensive reviews see [1–3]). In general, intrinsic prezygotic isolation is not very common in yeasts, with many species readily forming hybrid zygotes in the lab [4,5] although extrinsic prezygotic isolation, in the form of geographic and ecological isolation, may act. Postzygotic isolation, acting after gamete production, is common and manifests as either inviability or sterility. Recent work has shown that intraspecific and inter-specific postzygotic isolation in yeast can be driven by combinations of nucleotide differences, such as sequence divergence causing anti‐recombination and genetic incompatibilities between pairs of alleles on the nuclear or mitochondrial genomes, or by large-scale chromosomal changes, including chromosomal rearrangements and differences in ploidy, producing unbalanced gametes.

**Figure 1:**
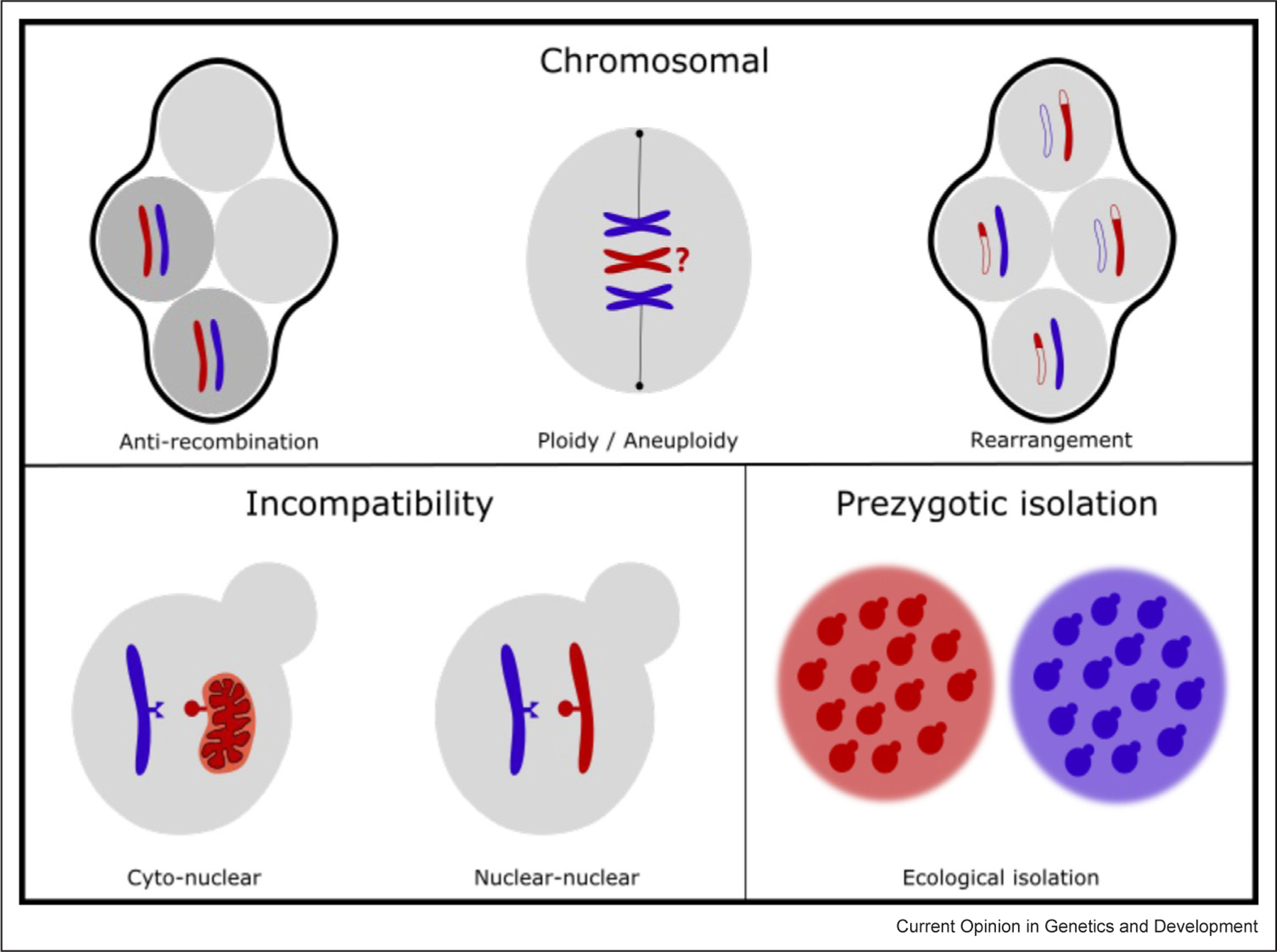
Mechanisms of reproductive isolation in yeast. The main reproductive barriers in yeast can be broadly broken down into three categories: those that affect whole chromosomes, those that are caused by genetic incompatibility at a few loci, and prezygotic isolation. Anti‐recombination inhibits correct segregation of chromosomes during meiosis, resulting in spores that carry either two copies or zero copies (inviable) of the chromosome. Both polyploidy and aneuploidy can result in difficulties segregating chromosomes evenly during meiosis. Chromosomal rearrangements, such as the reciprocal translocations represented here, lead to unbalanced gametes missing genes in translocated regions. Genetic incompatibilities can act either between nuclear genes and mitochondrial genes or between sets of nuclear genes. Finally, prezygotic isolation in the form of ecological isolation can occur when different genotypes are able to thrive under different environmental conditions.

On the surface, it appears strange to use yeasts, organisms with alternating sexual and asexual life cycles, to study an inherently sexual property. However, the genetic mechanisms of RI in yeast are the same as those in obligately sexual organisms, such as plants and animals (Figure 1). Furthermore, the availability of thousands of strain isolates, an inducible sexual cycle, and the ability to utilize experimental evolution makes *Saccharomyces* yeasts an excellent model system to study the early and late stages of the speciation process [6]. Since most research on RI is concentrated on *Saccharomyces* yeasts, this will be our primary focus as well. Among yeasts more broadly, not much is known about RI [1], but we will touch on some recent studies where relevant.

### Prezygotic isolation

Many yeast species are able to hybridize in the lab but are potentially ecologically isolated in nature. Mating genes allow hybrid zygote formation among species of many yeast genera, including *Saccharomyces* [4], *Cryptococcus* [7], and *Hanseniaspora* [8]. A notable exception is observed in the pheromone/receptor system of *Schizosaccharomyces pombe* and its close relative *S. octosporus* [9,10]. These results, along with experimental studies in *Saccharomyces* [4,11], demonstrate the potential for spores of *Saccharomyces* species could be attributed to differences in germination timing instead of species recognition [12,13]. Recent work by Plante and Landry (2021) found that some lineages of *S. paradoxus* also have different environmental requirements for spore activation and germination [14]. Differences in germination conditions and timing between species are forms of allochronic isolation, where the development of non-overlapping mating periods and therefore mate availability drives RI in sympatry.

There is also increasing evidence for ecological isolation at the vegetative life stage, by which different species or different populations are not active in the same space at the same time (Figure 1). Spurley et al. (2022) sampled nearly 2 000 yeast isolates from a broad spectrum of taxa and identified a number of taxon–substrate and plant genera associations [15]. These associations provide fine-scale geographic isolation of species, potentially preventing hybridization. In extreme examples of host-plant associations, such as in the plant pathogen genus *Taphrina*, there is strong phylogenetic congruence between the yeasts and their hosts [16]. In addition, there is a strong effect of isolation temperature on the phyla and species of yeasts recovered from natural samples [15] and physiological profiles differ between species of *Cryptococcus* [7]. When Bleuven et al. (2019) competed a collection of 550 barcoded strains of *S. paradoxus*, which included diverged lineages, they found extensive genotype-by-environment interactions for fitness [17]. All of these differences in ecology and geography further isolate species, even in sympatry, allowing for genetic differences to accumulate.

### Anti‐recombination and chromosome segregation

The primary reproductive barrier known to act in interspecific yeast crosses is the activity of anti-recombination proteins in response to sequence divergence. This barrier is best studied in the hybrids of the sibling species *Saccharomyces cerevisiae* and *S. paradoxus*, which have 12% SNP-level distance [18]. According to the anti-recombination model, genome-wide nucleotide mismatches suppress meiotic crossovers, which are essential for proper chromosome segregation, thus rendering hybrid gametes inviable due to at least one missing chromosome (Figure 1). The recent work by Rogers et al. (2018) showed that meiotic chromosome segregation in hybrids is nearly random across all chromosomes, explaining 97.3% of the observed hybrid sterility [18]. In addition, more recent work by Bozdag et al. (2021) provided the most direct evidence for the major role of anti-recombination in hybrid sterility by meiotically repressing two anti-recombination proteins (*MSH2* and *SGS1*) [19]. As a result of improved recombination and chromosome segregation, hybrid gamete viability increased by 64-fold, from 0.46% to 32% [19], reaching levels seen in some intraspecific crosses (e.g. [20]).

**Figure 2:**
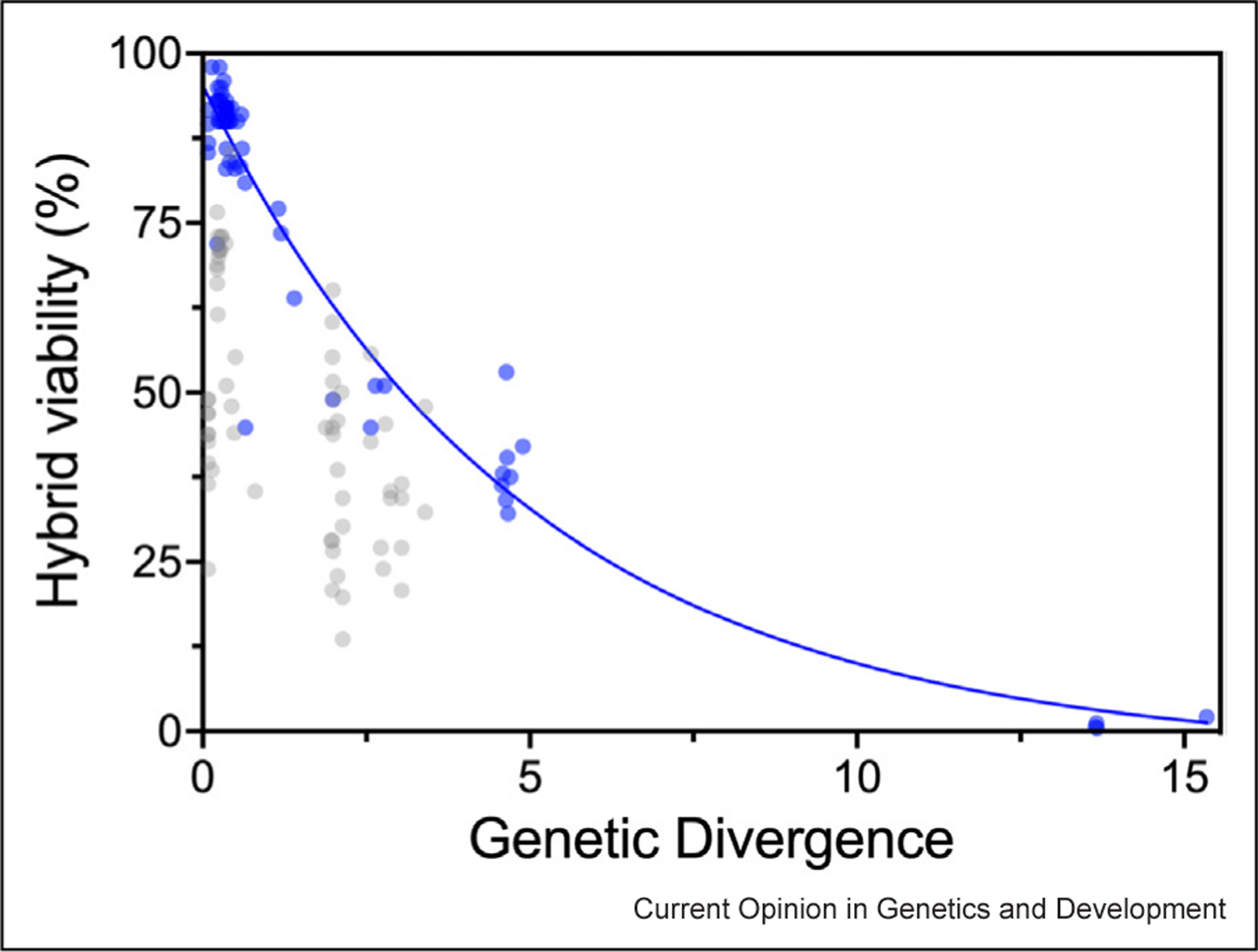
Genetic distance results in an exponential decay in hybrid gamete viability. Genome-wide sequence divergence results in a correlated exponential decline (blue line; R^2^=0.9307; df=66) in viability of hybrids with collinear genomes (blue symbols). This negative impact is noticeable even at divergence levels < 2%, highlighting the role of anti‐recombination in the early phases of the yeast speciation process. On the other hand, large-scale chromosomal differences constitute a powerful part of RI across many incipient species crosses but not between established species (gray symbols — excluded from the correlation analysis). Data for this figure are extracted from [13–15,35,76] and are available in Supplementary Data 1.

Although the importance of anti-recombination for intraspecific crosses tends to be dismissed, analysis by Rogers et al. (2018) showed that even at 1.4% SNP-level distance, chromosomes of an *S. paradoxus* hybrid cross fail to properly segregate on average 3.4% of the time, and as much as 10–14% for small chromosomes, killing a quarter of all gametes [18]. Furthermore, in *S. cerevisiae* crosses with only 0.7% SNP-level distance, anti-recombination protein activity reduces genome-wide crossover events by 20% [21], partially explaining why there is a negative correlation between divergence and gamete viability (fertility) that starts acting at the incipient-species level (Figure 2, blue symbols). Finally, because crossing over starts with a homology search across chromosomes in many taxa [22], DNA polymorphism can be inherently important to the efficiency of recombination in any organism. Indeed, it has been shown that a decline in sequence homology results in an exponential decline in the recombination rate in distinct lineages of prokaryotes and eukaryotes [22,23], analogous to the exponential decline observed in collinear yeast hybrids (Figure 2). While not necessarily attributable to anti-recombination, this pattern implies a broader — and potentially more nuanced (e.g. [24]) — role for sequence divergence in impacting genetic mixing across taxa [23,25–28]. Nevertheless, the question of whether genetic divergence affects chromosome segregation in species outside of yeast is yet to be studied more extensively.

### Genetic incompatibilities

The Bateson–Dobzhansky–Muller model of genetic incompatibility attributes postzygotic RI to negative interactions between alleles diverged at two or more loci between species’ genomes (Figure 1). There is evidence for two-locus incompatibilities across eukaryotic taxa from fruit flies to plants to fish species (e.g. [29]), but their significance for RI in yeast is not well known. While negative interactions can be widespread between the mitochondrial genomes of different yeast strains (i.e. mito–mito epistasis) [30], here we focus on recent work that genetically mapped two-locus incompatibilities between nuclear loci or, more importantly, between nuclear and mitochondrial loci.

### Nuclear–nuclear incompatibilities

Intraspecific crosses of natural or experimentally evolved yeast populations show that genetic incompatibilities readily evolve between pairs of interacting loci located in nuclear genomes and negatively impact hybrid gamete viability [31–33]. Recently, examining an intraspecific cross of two *S. cerevisiae* strains with 0.35% genetic distance, Hou et al. (2015) discovered a condition-specific incompatibility between a tRNA suppressor gene (*SUP7*) and a nuclear-encoded mitochondrial gene (*COX15*), showing that 25% of the hybrid segregants were respiratory deficient [34]. Other than mapping this specific two-locus interaction, they note that most (103 out of 117) of the negative interactions involve more than two loci (i.e. complex interactions). Finally, their study calls for more work testing for incompatibilities across diverse environments [34], as potentially lethal incompatibilities can be masked under standard laboratory conditions.

So far, there is no evidence for lethal two-locus incompatibilities between yeast species. Promisingly, by dissolving the anti-recombination barrier in an interspecific cross of *S. cerevisiae* and *S. paradoxus*, we were able to detect four putative incompatible loci between the two species’ nuclear genomes [35]. This and similar analysis by our colleagues [36,37] indicate the potential of partially lethal incompatibilities involving more than two loci, but in all these cases it is imperative to experimentally validate the identities and effects of the incompatible alleles.

### Mitochondrial–nuclear incompatibilities

Even though *Saccharomyces* yeast can generate energy through fermentation and thus grow without aerobic respiration, mito-nuclear incompatibilities can form a partial reproductive barrier because meiosis and gamete production cannot proceed without respiring mitochondria. Jun-Yi Leu and colleagues successfully mapped mito-nuclear incompatibilities in interspecific crosses of yeast. They showed that impaired interactions between nuclear genes and mitochondrial mRNA lead to a reduction or inhibition of Adenosine triphosphate ATP synthesis through the mitochondrial electron-transport chain, rendering some hybrid segregants partially or fully sterile [38,39]. Most recently, Jhuang et al. (2017) discovered an incompatibility between an RNA-binding protein (COB and COX1 mRNA maturation *CCM1*, chrVII in *S. bayanus*) and a 15S-rRNA (mtDNA in *S. cerevisiae*), causing a mild respiratory defect in hybrid gametes [40]. Furthermore, their analyses indicate that genes involved in this incompatibility show rapid evolutionary change across species and taxa, potentially forming incompatible interactions across yeast and some plant species [40].

### Large-scale chromosomal differences Rearrangements

Chromosomal rearrangements, such as translocations and inversions, can negatively affect the viability of hybrid offspring. For example, a reciprocal translocation present in only one of two parents will result in offspring who do not carry a complete set of genes (Figure 1). Simultaneously, inversions can inhibit recombination in a certain region or, if recombination does occur, generate unbalanced gametes. There is ample evidence for chromosomal rearrangements playing an important role in RI within species of yeast (*S. cerevisiae* [41], *S. paradoxus* [20], and *Schizosaccharomyces pombe* [42]) but limited evidence of them being significant at the interspecific level in *Saccharomyces* yeasts [43–46]. Correspondingly, there is no more variation in chromosomal arrangement between species than there is within.

There is not much recent work in this area, but there is evidence from lab experiments with *S. cerevisiae* that genomic instability can affect the entire genome systematically, leading to chromosomal alterations, including loss of heterozygosity (LOH), aneuploidy, and translocations from nonallelic homologous recombination [47], potentially leading to RI between differently affected strains. In addition, chromosomal rearrangements, including inversions and a reciprocal translocation, have been found among *Cryptococcus* species, but it is difficult to assess their importance for RI without follow-up studies [7]. An investigation of lab-induced translocations in *C. neoformans* found a large effect on sexual reproduction with no effect on vegetative growth, indicating a potentially large role for translocation in RI in this genus [48].

### Ploidy

While all *Saccharomyces* yeasts have the same base number of chromosomes (16) and ploidy (2n) [2,44], ploidy changes have the potential to play a large role in their evolution. *Saccharomyces* yeasts are thought to have an ancient allopolyploid origin with whole-genome duplication (WGD) occurring about 100–150 million years ago [49,50]. In modern *Saccharomyces* yeasts, polyploid isolates (3–5n) are common among domesticated strains [6,51–53] and have been linked to hybridity [51].

Ploidy changes can cause reproductive isolation [51]. When a tetraploid gamete (2n) fuses with a diploid gamete (1n), they form a triploid (3n), which has problems during meiosis and produces unbalanced gametes (Figure 1). Indeed, beer strains, usually triploid or tetraploid, tend to have low sporulation efficiency and spore viability [53]. When comparing interstrain hybrids, Charron et al. [2019] found that triploid hybrids had even lower spore viability (20.7%) than their diploid counterparts (37.6%) [54].

### Aneuploidy

Like chromosomal rearrangements and ploidy, variation in chromosome number is not observed between species of *Saccharomyces* yeast. Within species, however, there is frequent aneuploidy (deviation from a multiple of 16 chromosomes) in some lineages, especially those from clinical and industrial niches. The emergence of aneuploid yeast may be tied to genomic instability, which itself is linked with both polyploidy and hybridity [54–56]. This is an interesting case in which hybrid fitness can decrease, and therefore RI increase, without further generations of mating, an alternative to traditional hybrid breakdown. Recent work has shown that within *S. cerevisiae*, the prevalence and fitness costs of aneuploidy vary by genetic background [57]. The different tolerances for aneuploidy between strains can help generate RI as matings between types will lead to mismatches between chromosome copy number and genetic background.

The costs associated with aneuploidy can occur at both the vegetative and meiotic life stages. First, gene imbalances (extra copies of some genes) are costly during vegetative growth in some strains. Evidence for this comes from natural strains, in which the frequency of chromosome amplification is negatively correlated with chromosome size and covarying gene content [57], and from lab experiments [58,59]. Second, aneuploidy is thought to cause problems in sporulation efficiency and spore viability due to defects segregating aneuploid chromosomes and high frequencies of deleterious alleles, which are masked when heterozygous but can cause problems when exposed to selection in spores [53,60,61]. De Chiara et al. (2022) found meiotic progression to be slower in aneuploid strains compared with diploid strains, especially when there were multiple aneuploidies present [61]. In a large-scale survey of *Saccharomyces cerevisiae* lineages, initial evidence indicates that both the ability to sporulate and the number of spores formed (four vs. two) associated with chromosome amplification appears to be clade-specific, but aneuploidy is more common among strains that have lost efficient sporulation [57].

### How hybrids can overcome reproductive isolation

Considering the strength of these postzygotic barriers, especially at the interspecific level, how can we explain the discoveries of genomic introgressions and a growing number of yeast hybrids? It may be that the yeast life cycle, which alternates between mitotic (asexual) and meiotic (sexual) reproduction, allows hybrids to resolve sexual barriers during asexual growth.

Genetic incompatibilities involving few loci, whether they be nuclear–nuclear or mito‐nuclear, typically only affect some of the hybrid segregants, allowing the rest of the population to survive. Respiration is a common theme among all known incompatibilities, affecting both growth in respiratory environments and the ability to undergo meiosis. Strong selection for subpopulations lacking these incompatibilities, followed by multiple rounds of vegetative growth, can allow certain hybrid genotypes to thrive and be meiotically competent [62,63].

The anti-recombination barrier can be overcome by restoration of homologous pairing, and therefore recombination, during meiosis. Homologous blocks of the genome are generated through mitotic LOH events, which can be widespread if there is a lot of genomic instability, as has been inferred for the *S. cerevisiae* Alpechin lineage with abundant *S. paradoxus* introgressions [64]. Even without LOH, rare viable hybrid spores can be produced (~1%). Owing to their rarity, they are very unlikely to mate with each other, but they have the potential to switch mating type and mate with their daughter cells, breaking down the anti-recombination barrier completely, or mate with parental species, reducing the barrier by 50% with each subsequent backcross. Similarly to mating-type switching, WGD of a hybrid provides each chromosome with a nondiverged pair during meiosis, a tool that has been used experimentally [65], and a phenomenon that was recently observed to occur under relaxed selection in the lab [54]. Following up initial observations, Marsit et al. [2021] found spontaneous WGD in up to 11% of populations within 770 asexual generations [55]. The tetraploids were found to have both parental genomes entirely duplicated, likely through endoreduplication, and most had significantly increased spore viability. Rates of WGD have the potential to be even higher in natural strains capable of switching mating types where a single diploid cell with a damaged mating-type locus could produce progeny of the opposite mating type and mate to form a tetraploid.

However, even when allotetraploid hybrids make viable spores, these spores cannot mate, this is known as the second sterility barrier (after anti-recombination) [66]. The diploid spores of allotetraploids will be heterozygous at the mating locus, suppressing both mating and mating-type switching. Because all *Saccharomyces* species, and many other yeast species, seem to have compatible mating loci [4,5,7,8], this is a problem in all interspecific crosses attempted [66]. Again, this barrier can be broken during asexual growth by damage of the mating locus or loss of the chromosome carrying it, as has been observed in *Zygosaccharomyces* [67,68] and *Saccharomyces* [66], respectively, as well as by mitotic LOH at the mating locus.

Large-scale chromosomal changes such as rearrangements and aneuploidy can also be resolved during asexual mitotic growth. The same genomic instability that generates these changes can also reverse them. In particular, polyploid and aneuploid genomes can revert to a diploid state through chromosome loss, as has been observed [55,69]. Such reversion would break the ploidy-based reproductive barrier but could create a strain that is isolated from its parents based on chromosomal composition and anti-recombination, making further meiotic reproduction difficult. This is reminiscent of the parasexual cycle observed in *Candida* species, by which genetic variability is increased through the fusion of diploids to form a tetraploid followed by concerted chromosome loss back to a diploid state [70]. The pathogen *C. albicans*, as well as other species in the genus, seems to have either a hybrid origin (e.g. [70–72]) or current hybridization [73]. Many of these hybrids are likely unable to undergo a normal sexual cycle, including meiosis [70], and have undergone most of their evolution asexually. In general, while resolution of meiotic RI is possible, and perhaps even common at the early stages of hybridization, most long-surviving interspecific hybrids in *Saccharomyces* are isolated from fermentative environments [3], where yeast grows primarily through asexual mitotic divisions seemingly successfully avoiding the problems of meiotic reproduction.

## Conclusions

There are many paths to reproductive isolation available to yeast. Ecological and geographic isolation allows all types of genetic differences to accumulate among yeast populations. Genetic incompatibilities, both nuclear–nuclear and mito-nuclear, may act within and between species of yeast, but these seem to often be environment-dependent. Interestingly, these may be environment-dependent because they have evolved in response to differential environmental adaptation between types (e.g. [37,74,75]). Large chromosomal differences (rearrangements, polyploidy, and aneuploidy) play a widespread role within species of *Saccharomyces*, but interestingly, they are not fixed between species. Finally, anti-recombination is a major driver of RI, both within and between species. In Figure 2, we see that even at low levels of genetic divergence (within species), hybrid viability decays exponentially with genetic divergence. In this way, each individual SNP difference between strains can be considered as a small genetic incompatibility, contributing to anti-recombination.

Despite everything we know about RI in yeast, there is still much to be learned. Among the best-studied genus, *Saccharomyces*, it is unclear why some barriers, such as genomic rearrangements and aneuploidy, appear within species but are not fixed between them. Could it be that these barriers are transient, getting resolved during periods of asexual growth? Meanwhile, anti-recombination is important for both intraspecific and interspecific RI. Could adaptation speed up the accumulation of genomic differences, and therefore anti-recombination, between isolated strains? Finally, hybrid lineages have been very successful in many yeast genera, such as *Saccharomyces* and *Candida*, circumventing meiosis altogether in favor of a purely asexual or parasexual lifestyle. How important is this history of hybridization for the evolution of new yeast species? In answering these questions, we are bound to reveal interesting insights into the lifestyles of these workhorse microbes as well as general molecular mechanisms of reproductive isolation.

## Supplementary Material

Supplementary Material
